# Telemedicine and the standard of care: a call for a new approach?

**DOI:** 10.3389/fpubh.2023.1184971

**Published:** 2023-05-04

**Authors:** Tomáš Holčapek, Martin Šolc, Petr Šustek

**Affiliations:** ^1^Department of Medical Law, Faculty of Law, Charles University, Prague, Czechia; ^2^Department of Civil Law, Faculty of Law, Charles University, Prague, Czechia

**Keywords:** telemedicine, remote health services, standard of care, legal liability in health care, public health, health law

## Abstract

Telemedicine, understood as the provision of health care by a health professional to a patient who is physically not in the same location as the health professional, has many actual and potential benefits. It also has some disadvantages though, including a higher risk of misdiagnosis or another unfavorable outcome of certain remotely-provided services. In principle, the regime of legal liability for medical malpractice is the same for telemedicine as for traditional physical care. The general outline of the standard of care, which includes respect for medical science, the patient's individuality and objective possibilities, is abstract and flexible enough to be used for remote care without the need for redefinition. The quality of health care should be evaluated on the basis of the whole scale of risks and benefits it brings to a particular patient, including accessibility and comfort. In general, it should be permissible to provide a medical service remotely on the condition that its overall quality is at least as good as its comparable physical alternative. In other words, certain decrease in quality of some aspects of remote care can be compensated by other advantages. In terms of public health, support for telemedicine may bring a great improvement in the access to health care, and thus help significantly the individual members of the population. From the individual perspective, respect for personal autonomy implies that a patient should have every right to opt for a remote service, provided that there exists a true choice between meaningful options which is made on the basis of full information. If telemedicine is to fulfill its potential without sacrificing the protection of patients and their rights, reasonable guidelines for remote services need to be defined for particular medical fields, and for specific procedures within them. Among other issues, these guidelines must address the question of when it is necessary to refer the patient to physical care.

## 1. Introduction

Telemedicine has been a widely discussed topic in recent years, especially since the onset of the COVID-19 pandemic (1, 2). Digitization of health care is on the rise (3). Demographic changes (4, 5) and technological developments (4, 6) put a strain on the financial and human resources of health systems. New methods of diagnosis and treatment promise great benefits, yet at the same time, they make it difficult to maintain access to quality care for the general public. These powerful factors will almost certainly increase the importance of telemedicine in the foreseeable future.

If understood as remote provision of health services, telemedicine has been practiced for more than a century. In November 1879, just 2 years after the invention of telephone, Lancet published a mention about a physician who had been able to avoid an unnecessary midnight home visit by organizing care for a child with suspicious cough over telephone (7). Today, telemedicine is a much more sophisticated and broad field, ranging from the remote provision of medical advice similar to that in the 19^th^ century (only via internet, instead of telephone) to high-tech continuous monitoring of vital functions, or the involvement of artificial intelligence (machine learning and deep learning systems; hereinafter “AI”). Its potential is accompanied by various problems though, including data protection (8), bias in data-driven AI systems (9, 10), and others. All these developments and challenges have an impact on the expected standard of care, and may give rise to legal liability should the care provided fall below this standard.

Some writers suggest to define a “new form of malpractice” for telemedicine (11), while others do not consider such a dramatic measure necessary (12). Arguably, there is no reason to completely remodel the common basic elements of legal liability, such as a breach of legal duty, the existence of harm, and a causal link between the two. What needs to be discussed, however, is their interpretation and actual application in cases involving telemedicine [see Koch (13) for a similar line of thought with respect to the Principles of European Tort Law]. In medical malpractice, the critical issues are typically whether the health service provider's conduct complied with the standard of care and whether it led to any harm suffered by the patient. The introduction of new technology, especially advanced software solutions and artificial intelligence, could make it particularly complicated to prove these elements of liability in a lawsuit. Some legal theorists (14) and legislative proposals, such as the recent proposal for an AI Liability Directive at the level of EU (15), aim to solve these problems by various legal techniques including rebuttable presumptions of non-compliance and causation, applicable in certain circumstances. But we shall leave procedural aspects aside in this paper, and rather focus on the effect of telemedicine on the content of standard of care.

## 2. The concept of telemedicine

The nomenclature for digital and remote health services has yet to be standardized. Nevertheless, several categories are usually distinguished. The following classification (16) offers a suitable set of definitions:

- **eHealth** is the broadest category and encompasses all systematic use of information and communication technologies (ICT) in health care. It serves to augment and connect every aspect of health care including the support of preventive care, diagnostics, treatments, and health care administration.
- **Telehealth** is a subcategory of eHealth. It denotes any efforts to prevent an illness and protect health via ICT means. While telemedicine focuses on clinical applications, telehealth also encompasses tools for education and promotion of a healthy lifestyle.
- **Telemedicine** is a subcategory of telehealth. EU authorities define telemedicine as “*the provision of healthcare services, including remote care and online pharmacies, through the use of information and communication technologies, in situations where the health professional and the patient (or several health professionals) are not in the same location”* (17). These services may include e.g., remote consultations between a health professional and a patient, telemonitoring of health and diagnostic parameters, data transmission to a specialist, and remote consultations among health professionals in respect of a particular case.

In this paper, we discuss the standard of medical care, which assumes by definition that it is applied in clinical settings. Hence, our focus is on telemedicine.

## 3. Standard of care

The standard for assessing the conduct of a health professional in a particular case may have a different definition in each legal system, but often includes the following three components:

- **Compliance with the rules of science and acknowledged medical procedures**. The objective aspect of the standard requires health care providers to comply with scientific evidence embodied in guidelines, recommended procedures, medical protocols, scientific studies and papers in medical journals etc. These sources can have a crucial role in the judicial assessment of the provider's actions. Their form and content is not identical in all countries, but the fundamental principles of the practice of medicine apply universally (18). In its article IV. C.−8:104, the Draft Common Frame of Reference (19) describes the required care and skill as such “*which a reasonable treatment provider exercising and professing care and skill would demonstrate under the given circumstances.”* An absolute consensus is probably unattainable in any profession. Hence, it is usually sufficient if a procedure is accepted by a relevant part of professionals in the particular field. For example, this principle was expressed succinctly in English common law decades ago in the judgment in *Bolam v. Friern Hospital Management Committee* [1957] 1 WLR 582 (“*he is not guilty of negligence if he has acted in accordance with a practice accepted as proper by a responsible body of medical men skilled in that particular art”*) (20).
- **Respect for the patient's individuality**. Diagnostic and curative procedures must sometimes be adapted to suit the patient's unique biomedical, psychological, social, cultural or religious needs. Ignoring the patient's specific context might render the care suboptimal and possibly cause harm. Blind adherence to guidelines or protocols may be negligent in itself (21).
- **Regard to particular conditions and objective possibilities**. Nobody is obliged to do the impossible (22). While health service providers must comply with the prescribed equipment and staffing requirements, it is inevitable that the means and equipment available at particular health facilities will differ. The number of staff on duty fluctuates (compare a shift on a workday to a weekend night). An extraordinary event, such as an outbreak of the COVID-19 epidemic, may cause periods of overload when the providers simply do not have enough resources for all the patients.

## 4. Applying the standard of care to telemedicine

On an abstract level, the three components of the standard of care described above can be applied to all types of health care, whether provided remotely or in traditional settings. It has been stated that telemedicine must be held against the same standards as physical care (23, 24). This also includes the ethical framework of telemedicine, which does not substantially differ from other clinical care (25, 26) and can be based on a modified theory of the four ethical principles of beneficence, non-maleficence, autonomy and justice (27).

This might not pose any problem: telemedicine already improves the quality of care in specific cases, as supported by primary evidence (28–33) as well as meta-analyses and systematic reviews (34–36). It may have further benefits in the future (37). In other situations, however, the physician is simply unable to utilize all the established procedures with respect to a remote patient. Some common steps, e.g., palpation or auscultation, are unavailable. An experienced physician is often able to discern a lot of information from the patient's locomotion and behavior, and may suspect a health issue even before the patient sits down in their office (38). These clues are likely to be neglected in the current practice of telemedicine. Physicians might then tend to mitigate the heightened risk of misdiagnosis (39) by overprescription of drugs (40).

These challenges should be reflected in education offered by medical schools (41, 42). In addition, unavailability of certain techniques in remote settings can be compensated otherwise. Artificial intelligence may ask the patients questions that could be omitted by a physician due to the lack of time they can spend interviewing a patient. In the case of certain illnesses, implantable devices may collect much more data and with a higher accuracy than is possible during an interview (43). Nevertheless, the physical constraints associated with telemedicine may still mean that care provided to a patient in a particular case remotely might be somewhat riskier or less effective than could be the case otherwise.

However, the overall quality of care should be evaluated on the basis of all its aspects including accessibility and comfort for patients, as these elements undoubtedly affect both its objective efficiency and perception by its recipients. The demand for health services has been increasing in modern world, which widens the gap between the need and the supply (4, 5). Telemedicine can help tremendously in addressing the problem of access to care, including shortened waiting times, partial substitution of professional workforce by AI (helping to resolve the problem of staff shortages), maintained availability of health care outside of large cities, and improved access to specialists and second opinions. The crucial question is whether the benefits that telemedicine might deliver with respect to the quality of health care, measured at the level of society as a whole, justify the use of a different standard for specific care provided remotely to a particular patient than that which would apply in other cases. In other words, the question is whether it is acceptable to interpret the abstract standard of care with such flexibility that telemedicine could be seen as complying with this standard even though it cannot utilize all the methods and techniques which would be available in traditional settings.

If this question is to be answered in the affirmative, the matter has to be considered from two points of view: public policy and individual autonomy.

### 4.1. Public policy and public health

The public policy aspect involves the promotion of public health, with the obvious line of reasoning being that implementing measures which improve health of the population as a whole will likely also benefit individuals. It is naturally not acceptable to use public health merely as a pretext for sacrificing the efficiency and safety of care for individual patients in order to obtain certain societal benefits. Such an approach would contradict the constitutional right to the protection of health in many countries. Similarly, Article 2 of the Council of Europe Convention on Human Rights and Biomedicine emphasizes the primacy of the interests and welfare of the human being over the sole interest of society (44). However, it may be in the legitimate interest of public health to modify (or interpret appropriately) the standard of care with respect to telemedicine in cases where it provides access to care that could otherwise be inaccessible (45). After all, complete unavailability of care would constitute a more serious violation of the right to protection of health than mere adaptation of its standard to allow for remote provision of care.

The legislator can use different tools to advance or suppress telemedicine. Regulating the standard of care is one of these tools, as it affects the willingness of health service providers to engage in this type of activity. Providers need to take potential legal liability into consideration, and as liability insurance is usually required, so do their insurers. Apart from liability considerations, the legislator's tools also include the powers to set the amount of reimbursements from the public health insurance system, and to regulate intellectual property rights and data protection. Telemedicine is helped by the processing (collecting, sharing, analyzing etc.) of large volumes of sensitive data. All stages of this process may be problematic from the perspective of privacy and cyber security. However, while data protection may arguably be connected with the standard of care in its broadest sense, it is a very complex issue on its own, beyond the scope of this paper (46, 47).

If providers are allowed to offer telemedicine, this also affects the relevant health service market. If they can specialize in telemedicine, i.e., without being simultaneously required to offer more traditional types of care, they can drive costs down and push less-specialized competitors out of the market. This, however, could then compromise the availability of health care. If, on the other hand, the legislator mandates that telemedicine can only be offered by providers who also operate traditional facilities, this might hinder the growth of remote services. These implications need to be carefully considered from the policy-making perspective.

### 4.2. Individual autonomy and informed consent

An important argument in favor of permitting telemedicine, even if it has certain disadvantages, consists in the patients' autonomy. A fully informed and competent patient is entitled to make almost any decision regarding their health care, including the refusal of life-saving care. A patient should arguably have the right to agree to a modified (and, in some aspects, lowered) standard applicable to remote care, and as a result, bear some of its heightened risks in exchange for greater accessibility, comfort or other benefits. If a remote service is a legally permitted alternative to traditional care, why should the patient not be entitled to choose between the two options? This is, of course, provided that the patient has been fully informed about their relevant advantages and disadvantages.

The patient's autonomy should not be routinely reduced to a mere choice between the two options (i.e., physical or remote care). Important clinical decisions should be reached via a shared decision-making process based on bilateral and continuous communication between the patient and the physician. This principle should motivate physicians to communicate meaningfully with patients and involve them in the guidance of their own care (48). Ideally, the patient should have the option to combine physical and remote services in a manner that optimizes the efficacy of care.

## 5. Adaptation of the standard of care for telemedicine

The standard of care, as outlined above on the abstract level, can be applied to telemedicine without any radical change. However, in order to have any practical value, abstract principles need to be translated into specific rules. Every procedure has its own specific standard in clinical practice. In addition, various ways to perform a certain procedure will usually differ in more than one aspect. If compared, each of them is often found to be better in some aspects and worse in others. The overall assessment is then based on the ratios of advantages and disadvantages. This distribution of benefits and risks may well be different in telemedicine as compared to physical care. The disadvantage related to the remote nature of care can be accepted if it is evened out–or even outweighed–by a certain advantage, such as better access to care or its greater comfort.

The ratio of advantages and disadvantages needs to be assessed with regard to every particular procedure. If no effort were made to strike the right balance, the principles applied would likely be too broad and cautious, such as “refer the patient to physical examination anytime there is a suspicion that remote service would not suffice to test all the possible diagnostic options.” Such heavy-handed rules would effectively stop the development of telemedicine. The providers of telemedicine would only facilitate the first contact with the patient, but would then almost always refer the patient to physical examination since they would not be able to comply with the specific requirements posed by standards which never anticipated the existence of remote care. In this way, telemedicine would become effectively useless, prolonging the patient's medical journey rather than making it easier and more comfortable. It is hard to imagine that there would be any relevant demand for telemedicine under such conditions. As a result, telemedicine would never be practiced in any relevant scope. The same would be true if excessively detailed guidelines were used, requiring certain particular steps that cannot be done remotely even if they can be functionally replaced by technology.

To make it possible for telemedicine to live up to its potential, bring maximum benefits to patients and help keep health systems functional and efficient, the medical profession will need to define field-specific and procedure-specific guidelines for remote care (49). While certain guidelines and recommendations have already been issued in some medical fields (50), their further elaboration and expansion are vital.

Typically, the guidelines need to define cases in which patients should be referred to physical care. If remote health services are accepted as a permissible option, their inherent limitations form part of objective possibilities to be taken into consideration when assessing whether the care provided complied with the legally expected standard. The provider should not be held liable for any harm suffered by the patient as a result of such limitations.

## 6. Discussion

The dawn of telemedicine must not impair the overall quality of care. Nevertheless, inherent constraints or lower performance in some particular aspects do not necessarily render telemedicine impermissible. The standard of care needs to be judged on the basis of the comprehensive risk-benefit ratio of each procedure with regard to each particular patient, and interpreted accordingly. Some disadvantages of telemedicine (the consequences of lacking physical contact between the physician and the patient) may be outweighed by its benefits, such as increased comfort, speed and accessibility for the particular patient, as well as maintained accessibility and cost sustainability of health care at the level of the health care system as a whole.

Similar trade-offs are known from the daily practice of medicine: guidelines can be modified or even not followed if this suits the needs of the given patient, even though such an approach might be riskier in certain ways. On a similar note, telemedicine needs to be assessed on the basis of the whole complex of its benefits and risks. With exceptions, such as the state of necessity, telemedical services always require free consent of the patient, who has been adequately informed about all the relevant benefits and risks of remote care as compared to care provided in the traditional way.

## Data availability statement

The original contributions presented in the study are included in the article/supplementary material, further inquiries can be directed to the corresponding author.

## Author contributions

All authors listed have made a substantial, direct, and intellectual contribution to the work and approved it for publication.
